# Expanding academic frontiers: a researcher’s experience in Health and Education exchange

**DOI:** 10.1590/0034-7167-2024-0530

**Published:** 2026-01-09

**Authors:** Rafaela Simon Myra, Sujay Saphire Galen, Aline S. Pagnussat

**Affiliations:** I Universidade Federal de Ciências da Saúde de Porto Alegre. Porto Alegre, Rio Grande do Sul, Brazil.; II Georgia State University. Atlanta, Georgia, United States of America.

**Keywords:** Cultural Competency, Health, Education, Fellowships and Scholarships, Health Care Economics and Organizations, Intercambio de Investigadores, Política de Educación Superior, Becas, Política de Intercambio de Investigadores, Competencia Cultural

## Abstract

**Objectives:**

to share an experience in the Institutional Doctoral Degree Program Abroad and to stimulate the interest of doctoral students in Brazilian graduate programs to undertake this type of internship.

**Methods:**

a descriptive analysis of the academic exchange experience was conducted during the period from November 2023 to June 2024.

**Results:**

the academic exchange fostered significant collaboration between the Federal University of Health Sciences of Porto Alegre and Georgia State University, resulting in the development of three potential collaborative publications. Additionally, the experience facilitated knowledge exchange and expanded intercultural communication competencies, strengthening academic ties and global research networks.

**Final Considerations:**

through collaborative exchanges, we pave the way for the continuous advancement of knowledge, technological innovation, and comprehensive socio-economic development. We hope more students will be motivated to engage in the global landscape of scientific research, thus expanding opportunities for growth and excellence for students and researchers.

## RESUMEN

**Objetivos:**

compartir la experiencia del Programa de Doctorado Sándwich en el Extranjero y fomentar el interés de doctorandos brasileños en este tipo de prácticas.

**Métodos:**

análisis descriptivo de una experiencia de intercambio académico entre noviembre de 2023 y junio de 2024.

**Resultados:**

el intercambio fomentó una significativa colaboración entre la *Universidade Federal de Ciências da Saúde de Porto Alegre* y la *Georgia State University*, generando el desarrollo de tres posibles publicaciones colaborativas. Además, facilitó el intercambio de conocimientos y amplió las competencias de comunicación intercultural, fortaleciendo lazos académicos y redes de investigación globales.

**Consideraciones Finales:**

los intercambios colaborativos impulsan el avance del conocimiento, la innovación tecnológica y el desarrollo socioeconómico integral. Se espera motivar a más estudiantes a participar en la investigación científica global, ampliando así las oportunidades de crecimiento y excelencia para estudiantes e investigadores.

## INTRODUCTION

Globalized education and high-yield research rely on mobility, collaboration, and internationalization. Continuous learning solidifies through practical experiences and interaction across different environments and people. The learning process also develops more effectively through observation and interpretation rather than passive reading or listening. Mobility facilitates contact and the exchange of experiences, reducing the distance between partner researchers and promoting closer and more productive collaboration^(1)^.

Since the 1990s, the number of international collaborations at the global level has increased. Recent data suggests that the percentage of scientific studies published in partnership rose from 16.7% to 21.7% between 2006 and 2016^(2)^. Historically, the highest levels of connections occurred between the United States of America (USA), Western European countries, and Japan. Currently, other research centers are emerging, such as those in the Asia-Pacific region and, to a lesser extent, countries like India and Brazil^(2)^. Publications with one or more international partners have a higher positive impact^(3)^, highlighting the importance of mobility initiatives. Knowledge exchange is an essential component in advancing global science.

In this context, it is important to consider the Latin American position within the international scientific collaboration. Studies highlight that collaboration with international authors, especially those from the Northern Hemisphere, is correlated with higher-impact research^(3)^. However, despite the benefits of these collaborations, there remains a disparity in the recognition of Latin American scientific contributions in prestigious journals. Articles from region tend to receive fewer citations despite their quality^(4)^. For example, the impact factor for non-collaborative articles from Latin America averages 66% of the overall impact factor of journals, whereas internationally collaborative articles tend increases those averages. This finding underscores the importance of international collaboration in increasing the visibility and recognition of research conducted in the region^(4)^.

Particularly in Latin America, collaborations between national and international universities have shown significant results. International collaboration enhances the scientific visibility and impact of research. This partnership not only enhances the quality of publications but also expands their reach and impact^(5)^. Furthermore, research with funding is associated with more international collaborations, perceived as an indicator of potential future academic success^(6)^, The interaction between funding and international collaboration underscores the importance of adequate investments and cooperation strategies to promote scientific excellence in the region

In Brazil, the internalization of postgraduate programs follows the guidelines of the Coordination for the Improvement of Higher Education Personnel (CAPES) and aims to boost Brazilian science. For this purpose, the Institutional Doctoral Degree Program Abroad (PDSE) was created to stimulate doctoral programs in Brazil and encourage the internationalization of science. This program seeks to improve the quality of student training by promoting research in advanced centers with different researchers and providing access to cutting-edge resources and infrastructure^(7)^.

Since 2011, the PDSE has increased the number of quotas granted to higher education institutions. The PhD scholarship costs less than full doctoral scholarships while still offering specialization in another country^(7)^. In Brazilian postgraduate programs, academic mobility is one of the main mechanisms for gaining international experience, forming networks, and establishing international research collaborations. It also enhances the capacity to analyze research objects, facilitates engagement with other cultures, and expands the worldview^(8)^.

The dissemination of these resources is vital to motivate more doctoral students to internationalize and promote Brazilian science. Therefore, the main objective of this article is to share an experience of international educational exchange in the PDSE PhD modality and to stimulate the interest of doctoral students in Brazilian graduate programs to undertake this type of internship

## OBJECTIVES

To share an experience of international educational exchange in the PDSE PhD modality and to stimulate the interest of doctoral students in Brazilian graduate programs to undertake this type of internship.

## METHODS

A descriptive analysis of the academic exchange experience was conducted during the period from November 2023 to June 2024

## RESULTS

### University environment experiences

During the exchange period, the activities focused on engaging in different enriching experiences. Among them, particular emphasis is placed on participating in research group activities at the motion technology laboratory. The research group focuses on developing outcome measures and assessment tools for clinical applications, including smart sensors and wireless devices, and analyzing human movement using 3D motion capture systems and electromyography. This immersion provided direct exposure to emerging technologies and methodological approaches used in scientific research. It also increased my understanding of how physiotherapy students are gradually introduced to the research process. Unlike more traditional methods, they were encouraged to independently formulate their research questions as they were introduced to new technologies within the lab. This approach allows students to conduct and think about the rationale behind each step of the research. Moreover, the exposure to emerging new technologies allowed me to see how teleassessments are becoming more frequent and how they are in the USA. Given the increasing use of remote care in the current global context, this experience served as an opportunity to stay aligned with recent advancements in digital health strategies.

It was also feasible to attend research conferences hosted by the GSU physical therapy department and the 9th Scientific Research Day of Lewis College. On this occasion, over 40 posters were submitted for evaluation, showcasing research conducted by students in the health sciences. This experience provided a platform to present the results of recent investigations by university research groups and innovations in health and technology. It was a valuable opportunity to listen to presentations and research discussions in different areas of health knowledge, such as nutrition and nursing. It also allowed social interactions with students from diverse backgrounds.

Another activity was the technical visit to Shepherd Center Hospital. It is a private, non-profit hospital specializing in medical treatment, research, and rehabilitation for individuals with spinal cord injury, traumatic brain injury, stroke, multiple sclerosis, spine problems, chronic pain, and other neuromuscular conditions. In addition to touring the hospital facilities, I observed the healthcare practices at the Andrew C. Carlos Institute for Multiple Sclerosis Care. I had the opportunity to exchange experiences and knowledge with local professionals and to observe their interdisciplinary approach. Physiotherapists, physical therapy assistants, occupational therapists, and speech therapists work together in treatments focused on continuing education, social activities, and individual and group therapy. Rehabilitation went beyond the clinical setting to include social activities and the development of a sense of community within the clinical environment. The hospital offers various programs to reintegrate individuals into the community - one example that stood out, distinct from the Brazilian context, is the support provided to U.S. military veterans. These programs focus not only on physical recovery but also on the social well-being of the participants, promoting group activities such as nature trails, mutual support, and social engagement.

Additionally, I became acquainted with and noted the differences in work and treatment approaches between Brazil and the USA. One of the main differences was a family housing facility connected to the hospital, designed to accommodate patients and family members. It allows families to stay close during inpatient treatment and outpatients to remain close to the hospital after discharge while continuing their treatment. These structures reflect a more comprehensive approach to patient care and support.

Moreover, this whole experience will lead to several significant scientific publications. It encompasses a systematic review, which is the primary focus of the scholarship, along with three potential collaborative publications. This is only possible due to the co-supervision agreement established by the institutions. Furthermore, I was invited to collaborate on a systematic review project led by a doctoral student in physical therapy at GSU. This represents a strengthening of the ties between researchers from GSU and UFCSPA, actively contributing to knowledge in physiotherapy. Also, those experiences will contribute to my academic growth, and further enrich my development as a researcher.

Finally, studying abroad offers more than quality academic training: it provides a transformative experience that encompasses personal and professional development on multiple levels. By immersing themselves in a new culture, students enhance their cultural competencies and gain a deeper understanding of the diversities and similarities among different peoples. Additionally, living in an international context fosters the development of empathy and interprofessional communication, as it values and respects different perspectives and histories in multicultural environments. As a result, global citizens with an expanded and more inclusive vision are formed, prepared to tackle global challenges with innovative and collaborative solutions^(9)^. This international experience broadened my worldview, and more importantly, reinforced my identity and development as an emerging researcher committed to global health advancement.

### The Institutional Doctoral Degree Program Abroad and its requirements.

The CAPES, under the Ministry of Education, created the PDSE program to boost academic training in Brazil. PDSE grants scholarships for students enrolled in doctoral programs to study abroad, allowing them to develop part of their research in partnership with a foreign institution. In addition to increasing collaboration and publications within the international academic community, the program also enhances access for the Brazilian academic community and brings greater visibility to scientific productions. Additionally, it exposes students to different academic methodologies and new management models in education from other locations^(7)^.

Students applying to participate in the PDSE can choose scholarships ranging from 4 to 12 months. Initially, the candidate must contact the foreign university and the professor with whom they wish to partner. However, it is encouraged that the contact between Brazilian students and foreign professors extends beyond individual interactions. By involving human resources but also their research laboratories, these connections are strengthened, ensuring their continuity and long term sustainability. This broader integration promotes a culture of collaboration and knowledge exchange, creating a solid foundation for mutual support between students and researchers, benefiting future candidates who may follow the same path of internationalization of academic research.

Another requirement to participate in the PDSE program is to demonstrate proficiency in the language of the destination country. The student must undergo a proficiency test and have a minimum ability to carry out their tasks in the destination country. Furthermore, students must create a detailed project outlining their planned study, demonstrate the importance of this research, and show a plan of activities. In this case, the plan of activities included exchanging experiences with researchers and healthcare professionals, field observations in reference hospitals in the city, participation in research group activities, the development of a systematic review, and a commitment to disseminate the international experience.

### Choice of country and university

Considering the factors of internationalization and opportunities for academic growth, we established a partnership with Georgia State University (GSU) in Atlanta, Georgia, United States of America (USA), and my home university, the Federal University of Health Sciences of Porto Alegre (UFCSPA), Porto Alegre, Rio Grande do Sul, Brazil. 

GSU played a crucial role in advancing social change and promoting equal opportunities in the Southern USA. Its history saw significant transformation, particularly regarding racial segregation in the USA. In 1956, Myra Payne Elliott, Barbara Pace Hunt, Iris Mae Welch, and other African Americans were denied entry to GSU, then known as the Georgia State College of Business Administration. This incident marked the beginning of desegregation at GSU. The university’s journey of social transformation serves as an example for other academic institutions, such as the University of Mississippi, which followed similar steps. Following this, GSU has committed to a socially conscious and inclusive approach. It has become a leader in diversified education and invests in implementing initiatives and programs that promote diversity and inclusion. As a result, GSU is one of the universities that graduates the highest number of African Americans in the USA. It demonstrates how a university can evolve from a reality marked by segregation to become an agent of positive change.

Currently, GSU’s history has outstanding academic rankings and indicators. It is particularly evident in the field of physical therapy. GSU’s physical therapy department is recognized as the oldest in Georgia and was the first to offer a doctoral program in this area. The program boasts state-of-the-art research facilities and technologies. Additionally, GSU has achieved remarkable records of students passing the American Physical Therapy Association exam, demonstrating the quality and excellence of the institution’s physical therapy program. On the other hand, UFCSPA, in Brazil, also offers a high-quality physiotherapy program. Despite differences in the model and approach of the physical therapy courses, both universities are recognized for technological excellence and commitment to training qualified professionals in the field. 

The partnership between UFCSPA and GSU began with the contact between the Research Group on Movement Analysis and Neurological Rehabilitation (GNeR) at UFCSPA and the motion technology laboratory at GSU. This relationship goes beyond individual and momentary collaboration. It was initiated with a Fulbright Scholar in Residence program during the 2022-2023 academic year, followed by a PDSE scholarship in 2024, and is expanding with future visits, lectures, and other potential exchanges between the two universities. Furthermore, as a strategy to further strengthen the relationships established during the PDSE program, it was decided to have the foreign professor as a co-supervisor for the whole doctoral program. This decision solidifies the ties and promotes closer and continuous collaboration between the institutions. With this, a more effective integration of knowledge and a higher number of international publications will arise. Through this partnership, both institutions seek to promote academic growth, exchange of experiences, and advancement in the field of physical therapy, contributing to the development of highly qualified professionals and the progress of science and society.

### Barriers and challenges

During this experience, we can encounter several challenges despite the valuable opportunity for professional growth that academic exchange represents. However, these barriers do not diminish the benefits; instead, they offer opportunities for learning and development. Adapting to a new cultural environment can be challenging. Differences in communication style, social norms, and academic practices may need help in initial integration. However, this experience also enriches cultural perspectives and enhances students’ intercultural communication skills.

When moving to another country, some factors need to be considered. Adapting to geographical distance, a new time zone and climate, and the experience of communicating in a new language can be challenging initially. However, this experience of living in a new country and continuously practicing another language significantly contributes to the development of independence and enhances linguistic skills.

Furthermore, one of the major factors contributing to low scientific output is limited access to funding opportunities and inadequate budgets^(10)^. The Brazilian government’s initiative to finance academic mobility projects is positive. However, even with this financial support, additional costs such as accommodation, food, and other personal expenses can be concerning and may exceed the amount provided by the scholarship. It is essential to manage resources efficiently to ensure the viability of the exchange program. Moreover, we need to bring light to this issue and advocate for more investments and resources for Brazilian students and education in general in Brazil. 

Despite the barriers and challenges, academic exchange is a highly rewarding experience. The program fosters an individual with a broader worldview and a greater understanding of global issues. This experience promotes the development of essential skills such as adaptability, problem-solving, independence, intercultural communication, and critical thinking. All these factors contribute to building more competent professionals. Therefore, even with obstacles, the academic exchange is valid, providing long-lasting benefits that positively impact the career and personal lives of the students involved.

## FINAL CONSIDERATIONS

The internationalization of Graduate Programs in Brazil aligns with CAPES’ goals to encourage and boost Brazilian science. It represents a step towards amplifying Brazilian scientific endeavors. Through collaborative exchanges, we pave the way for the continuous advancement of knowledge, technological innovation, and comprehensive socio-economic development. By disseminating such experiences, we hope more students will be motivated to engage in the global landscape of scientific research, thus expanding opportunities for growth and excellence for students and researchers.

## Data Availability

The research data are available only upon request.
